# A Critical Review on Immobilized Sucrose Isomerase and Cells for Producing Isomaltulose

**DOI:** 10.3390/foods13081228

**Published:** 2024-04-17

**Authors:** Wenjie Jing, Feihong Hou, Xinming Wu, Mingqiang Zheng, Yue Zheng, Fuping Lu, Fufeng Liu

**Affiliations:** Key Laboratory of Industrial Fermentation Microbiology, Ministry of Education, Tianjin Key Laboratory of Industrial Microbiology, College of Biotechnology, Tianjin University of Science & Technology, Tianjin 300457, China; jingwenjie@tust.edu.cn (W.J.); hou16631066554@163.com (F.H.); 22816791@mail.tust.edu.cn (X.W.); zmq3112@mail.tust.edu.cn (M.Z.); zhengyue-1986@hotmail.com (Y.Z.); lfp@tust.edu.cn (F.L.)

**Keywords:** immobilization enzymes, immobilized cells, isomaltulose, sucrose isomerase, green manufacturing

## Abstract

Isomaltulose is a novel sweetener and is considered healthier than the common sugars, such as sucrose or glucose. It has been internationally recognized as a safe food product and holds vast potential in pharmaceutical and food industries. Sucrose isomerase is commonly used to produce isomaltulose from the substrate sucrose in vitro and in vivo. However, free cells/enzymes were often mixed with the product, making recycling difficult and leading to a significant increase in production costs. Immobilized cells/enzymes have the following advantages including easy separation from products, high stability, and reusability, which can significantly reduce production costs. They are more suitable than free ones for industrial production. Recently, immobilized cells/enzymes have been encapsulated using composite materials to enhance their mechanical strength and reusability and reduce leakage. This review summarizes the advancements made in immobilized cells/enzymes for isomaltulose production in terms of refining traditional approaches and innovating in materials and methods. Moreover, innovations in immobilized enzyme methods include cross-linked enzyme aggregates, nanoflowers, inclusion bodies, and directed affinity immobilization. Material innovations involve nanomaterials, graphene oxide, and so on. These innovations circumvent challenges like the utilization of toxic cross-linking agents and enzyme leakage encountered in traditional methods, thus contributing to enhanced enzyme stability.

## 1. Introduction

**Figure 1:**
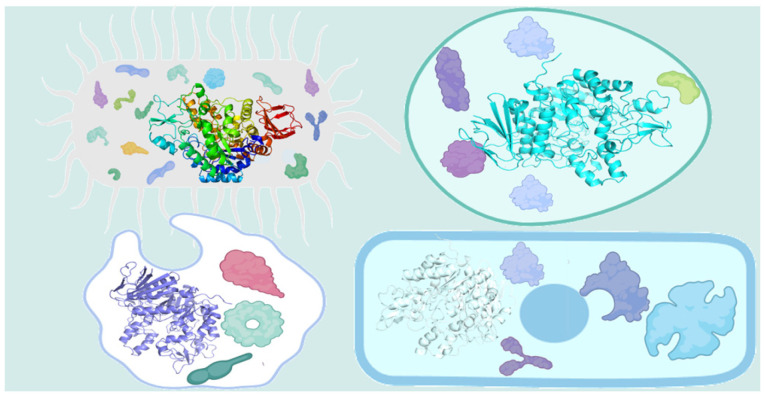
Schematic diagram of enzymes in a cell.

Enzymes are natural catalysts and belong to the category of biomacromolecules [1,2]. Currently, except that ribozymes are small RNA molecules with catalytic functions, most enzymes are fundamentally proteins (Figure 1). Enzymes possess characteristics such as high specificity, high catalytic activity, mild reaction conditions, non-toxicity, and environmental friendliness [3,4,5]. They are widely applied in industries such as food, agriculture, chemical engineering, pharmaceuticals, energy, and animal feed [6,7,8]. However, the disadvantages of biocatalysts, including susceptibility to inactivation and denaturation, high separation and purification costs, and low reusability, have hindered their further industrial utilization [9].

Sucrose is a commonly used sweetener in food and beverages [10]. Upon ingestion, sucrose is hydrolyzed into glucose and fructose, leading to an increase in blood sugar levels [11]. However, excessive consumption of sucrose can lead to obesity and an increased risk of heart disease and type II diabetes [12]. As people’s living standards and health awareness improve, there is a growing interest in seeking healthier alternatives to sucrose as functional sweeteners [13]. Isomaltulose, as a functional sweetener, is an ideal substitute for sucrose [14].

Isomaltulose (α-D-glucosylpyranosyl-1,6-D-fructofuranose) is an isomer of sucrose [15]. It is a reducing disaccharide formed by an α-1,6-glycosidic linkage between glucose and fructose [16] (Table 1). Its solubility in water is lower than that of sucrose, being 38.4% (*w*/*v*) at 20 °C, which is approximately half of the solubility of sucrose. As the temperature increases, the solubility of isomaltulose gradually increases, reaching 78.2% (*w*/*v*) at 40 °C and 133.7% (*w*/*v*) at 60 °C. Isomaltulose exhibits similar physical properties and sensory characteristics to sucrose [17], with a sweetness level approximately 50% that of sucrose [18]. Its melting point is 122 °C to 124 °C, which is considerably lower than sucrose’s melting point of 186 °C.

Isomaltulose has the following advantages including non-hygroscopic, acid stability, non-caries-inducing, prebiotic effects, and is suitable for diabetes. When mixed with citric acid under the same conditions, isomaltulose shows minimal increase in hygroscopicity, while sucrose’s hygroscopicity increases significantly. After 22 days of storage with citric acid, no other sugars are formed when isomaltulose is used as a sweetener in products containing organic acids, indicating its stability is higher than sucrose’s. In solutions of equal concentration, isomaltulose has lower viscosity than sucrose. It offers high stability, non-cariogenic properties, and benefits for fat oxidation and prebiotic effects [19,20,21]. In the small intestine, isomaltulose can be completely broken down and absorbed by the body. Compared to sucrose, isomaltulose is metabolized much more slowly, allowing blood glucose and insulin levels to remain relatively stable over time. This makes it suitable for consumption by individuals with diabetes, as it avoids rapid spikes in blood sugar and insulin levels [22].

Isomaltulose is a natural sugar that exists in trace amounts in products such as honey and sugarcane [23,24]. Its content is low and extraction is difficult, making it challenging to meet market demands. Therefore, in order to fulfill market needs, it is essential to enhance the production efficiency of isomaltulose. Currently, there are several methods for preparing isomaltulose, including microbial conversion, enzymatic conversion, chemical synthesis, and plant transgenic methods. Chemical synthesis is challenging, and plant transgenic technology is not yet mature, so the primary methods of production are microbial conversion and enzymatic conversion [25]. Microbial conversion mainly involves utilizing the sucrose isomerase in microbial cells to produce isomaltulose. This can be achieved through free cell or immobilized cell methods. Various microorganisms have been discovered that can convert sucrose to isomaltulose, including strains from the genera *Erwinia*, *Klebsiella*, *Serratia*, *Acinetobacter*, and *dispersed bacteria* [26,27,28,29,30,31]. Enzymatic conversion involves directly using sucrose isomerase to catalyze the isomerization reaction of sucrose, and isomaltulose is produced using free or immobilized enzymes. Both microbial and enzymatic conversion methods are based on the isomerization process of sucrose using sucrose isomerase. The α-1,2-glycosidic bond in the sucrose molecule is hydrolyzed within the enzyme’s active site and rearranged. The glucose monomer then forms an α-1,6-glycosidic bond with a fructose monomer (isomaltulose) or an α-1,1-glycosidic bond (trehalulose). These two isomers are released from the active site of the target enzyme. The broken α-1,2-glycosidic bond can also react with water molecules to form glucose and fructose (Table 1) [32,33].

Whole-cell biocatalysis directly converts sucrose to isomaltulose by using a live cell that can produce sucrose isomerase. This method is commonly cheaper than that of enzymatic conversion. Moreover, the stability of sucrose isomerase within cells is stronger than that of the free state. However, the additional consumption of sucrose is increased because sucrose serves as both a substrate and a carbon source for the cells. Additionally, these production strains do not have a food-grade genetic background, and immobilized cells have disadvantages such as cell autolysis and low product concentration due to cell metabolism [34]. The enzymatic catalysis method uses free sucrose isomerase or immobilized sucrose isomerase to catalyze sucrose to produce isomaltulose, which avoids some disadvantages of microbial direct conversion. Therefore, enzymatic conversion is receiving increasing attention. However, in enzymatic conversion, free enzymes are costly, prone to deactivation, and difficult to recycle. In contrast, immobilized enzymes can be recycled, exhibit good operational stability and storage stability, and are easily separated from products. Immobilized enzyme technology is continuously evolving, and there are various advanced techniques: carrier modification to enhance enzyme stability [35]; chemical modification to introduce specific groups for covalent binding to the carrier’s surface [36]; magnetic nanoparticle immobilization for easy recovery using magnetic properties [37]; cross-linked enzyme aggregates, which are easy to prepare and have high activity [38]; affinity immobilization for one-step purification and enzyme-specific immobilization [39]; metal–organic frameworks (MOFs) significantly enhancing enzyme stability [40]; nanoflower-based enzyme immobilization with simple operation and great application potential [41]; microwave-assisted immobilization techniques to overcome diffusion limitations in porous materials due to hydrophobic or hydrophilic properties [42]. As materials science, chemical engineering, biology, and related disciplines continue to develop, immobilized enzyme technology is constantly yielding new materials and techniques.

Both immobilized cells and immobilized enzymes have their own advantages and disadvantages. To achieve industrial production and reduce production costs, researchers have started to explore the use of immobilized cells/enzymes for isomaltulose production. They are seeking strains suitable for food production to undergo immobilization, investigating new methods for immobilizing enzymes, and developing novel carrier materials to enhance the stability and recyclability of sucrose isomerase. This ultimately aims to lower production costs. This review summarizes the improvements in immobilized cell/enzyme production of isomaltulose in recent years, both in traditional methods and through innovative approaches and materials.

## 2. Immobilized Cells

Immobilized cells are a commonly used method in industrial production, and they offer several advantages: (1) no enzyme separation and purification: immobilized cells eliminate the need for enzyme separation and purification, leading to cost reduction; (2) enhanced enzyme stability: enzymes within cells tend to have better stability compared to enzymes purified and isolated from cells; (3) simplicity of operation: the process of using immobilized cells is relatively straightforward; (4) no additional cofactors required: immobilized cells often do not require the addition of extra cofactors. In recent years, various methods involving immobilized cells for isomaltulose production have been developed, as shown in Table 2.

In recent years, researchers have explored various cell immobilization methods. At present, different methods of cell immobilization have some advantages and disadvantages, such as the following: sodium alginate embedding is non-toxic and harmless, easy to operate, but there are shortcomings of cell leakage. The composite embedding reduces cell leakage, but at the same time, reduces the cell contact rate with the substrate and reduces the conversion efficiency. However, the membrane reactor can reduce the cost and realize automatic production, but there are some disadvantages, such as uneven cell distribution and membrane fouling (Table 3).

### 2.1. Embedding Method

**Figure 2:**
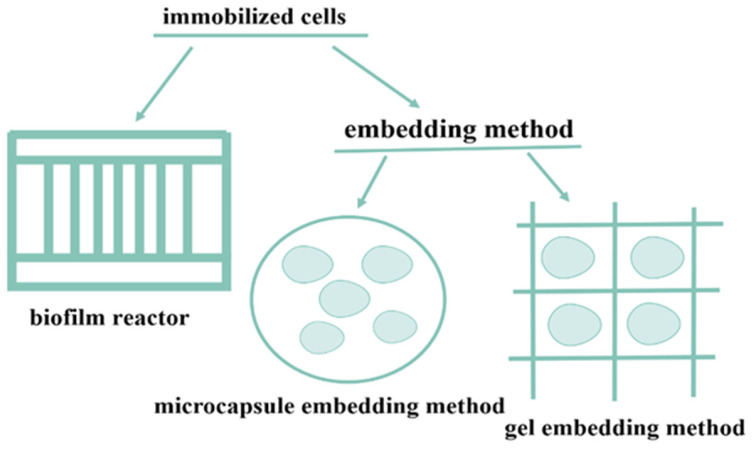
Schematic diagram of immobilized cells using the embedding method.

The most commonly used method of immobilized cells is the embedding method, which can be divided into the gel embedding method and microcapsule embedding method (Figure 2). The most commonly used method among them is to use sodium alginate as a carrier for embedding. This material is non-toxic and harmless, and the preparation is simple. After mixing the bacterial body with a certain concentration of sodium alginate solution, it is dropped into a certain concentration of CaCl_2_ solution to form microspheres, which can then be recycled. For example, Kim et al. thoroughly mixed *Serratia* sp. with a 3% sodium alginate solution and squeezed it dropwise into a 2.7% calcium acetate solution to form beads. This method is simple to operate, the spatial orientation of the enzyme is not disrupted, and the half-life is extended. After 35 cycles of use, the yield of isomaltulose is about 60%, which is currently the longest cycle [43]. To avoid the problem that endotoxin is derived from non-food-grade hosts and cannot be mass-produced sustainably due to poor thermostability, Hu et al. designed a dual mutant PdSI V280 L/S499 F derived from *P. dispersa* UQ68J through rational design and expressed it in food-grade Corynebacterium glutamicum cells, where the half-life of the enzyme was prolonged at 45 °C. Using 2.5% sodium alginate and 8.0% CaCl_2_, the conversion rate and mechanical strength were improved, and a one-step simplified immobilization method was used for fixation, which solved the need for cell permeation before immobilization, avoided cell lysis, and saved production time, more in line with industrial production. The maximum yield of isomaltulose in the cyclic reaction was 453.0 g/L, and after 26 consecutive catalytic reactions, the sucrose conversion rate still reached 83.2% [45].

Although sodium alginate has many advantages, it also has some drawbacks: low mechanical strength, cell leakage, and difficulty in recycling after swelling and crushing. Therefore, some researchers have adopted composite encapsulation and the use of cross-linking agents to solve these problems. For example, Su et al. used alginate to embed sucrose isomerase genes derived from *P. dispersa* UQ68J, which is a dual promoter recombinant strain with higher enzyme activity. When alginate is embedded on the surface and then coated with chitosan or polydopamine, the beads are more stable and durable. Additionally, this encapsulation-coating process enhances pH tolerance and slows the rate of enzyme activity loss at 50 °C. Using sugarcane molasses as a substrate to solve resource waste and environmental pollution problems, the yield of isomaltulose is 94%. After 30 cycles of recycling, the enzyme activity remains around 80% [44]. Souza et al. used 1.5% seaweed gum and 1.5% sodium alginate as embedding agents to embed *Erwinia* sp. D12. Seaweed gum is easily obtained and can save some carrier costs, but the highest yield of isomaltulose is only 61.94%. After 72 h of conversion, the yield of isomaltulose is 47.86%. When used continuously for 120 h, the yield of isomaltulose is notably low. This method can achieve a high conversion rate of sucrose to isomaltulose within 72 h [46]. In order to reduce cell leakage and improve the mechanical properties of the immobilized cells, the researchers utilized an embedding–cross-linking approach. Kawaguti et al. used sodium alginate as a carrier and glutaraldehyde as a cross-linking agent to immobilize *Erwinia* sp. D12. After optimization, it showed higher yield. After 282 h, the yield of isomaltulose was higher than 55% [47]. Oliva Neto et al. used sodium alginate as a carrier and glutaraldehyde and polyethylene imine as cross-linking agents to immobilize *P. rubrum*. The pellets remained active for up to 24 cycles. After 24 cycles of use, the yield of isomaltulose is still above 80% [48]. Carvalho et al. utilized sodium alginate–gelatin encapsulation for immobilizing *S. plymuthica*. They used transglutaminase as a cross-linking agent to enhance the texture of the polymer network, which resulted in a reduction in cell leaching. Sequential experimental design was employed to study the impact of variables on immobilization and conversion rates. The optimal experimental conditions determined were as follows: 1.7% sodium alginate, 0.25 mol/L CaCl_2_, 0.5% gelatin, 3.5% transglutaminase, and 33.5% cell aggregates. These conditions effectively improved cell immobilization and the efficiency of converting sucrose to isomaltulose, resulting in a yield of isomaltulose reaching 71.04% [49]. Souza et al. also used alginate–gelatin to embed *Erwinia* sp. D12, using transglutaminase as a cross-linking agent, and studied the effects of variables on immobilization and conversion rate using sequential experimental design. The optimal experimental conditions were 2.0% alginate, 2.0% CaCl_2_, 2.0% gelatin, and 0.2% transglutaminase. The highest yield was 327.83 g/L in the first 24 h, with an isomaltulose yield of 93.66%. Furthermore, a notable conversion activity was sustained during the initial 72 h [50].

### 2.2. Membrane Reactor

Membrane reactors can be divided into cyclic membrane reactors and integrated membrane reactors based on the different combination methods of reactors and separation devices. Integrated membrane reactors include hollow cellulose membrane reactors and composite membrane reactors. The membrane reactor has the following advantages: combining biological catalysis, product separation, concentration, and other operational steps into one step, reducing the cost of separation and purification in downstream processing; easy to continue and automate operations; the reactants can be removed in a timely manner, effectively avoiding product inhibition and side reactions; in the reaction, cells/enzymes can be reused, which can reduce production costs and improve production efficiency.

Membrane reactors offer advantages such as high production efficiency, simplicity of operation, and stability; thus, they have commonly been used to immobilize the cell and enzyme to produce isomaltulose. For example, Krastanov et al. utilized a hollow fiber membrane bioreactor to immobilize *S. plymuthica*. Using sucrose as the substrate, it lost only 11% of its activity after continuous operation for 90 days. However, the reactor was evaluated at 20 °C, and the implementation of a temperature control system would introduce greater complexity to the reactor design and consequently raise the production cost of isomaltulose [52]. Wu et al. fixed *Bacillus Subtilis* WB800-pHA01-palI in a biofilm reactor using a ceramic membrane as the separation system, and produced isomaltulose using pretreated sugarcane molasses as the substrate. After 12 cycles in the reactor, the sucrose conversion rate was over 90%. Sugarcane molasses is a by-product of the sugar industry. The use of low-cost sugarcane molasses as a substrate for the production of isomaltulose not only reduces production costs, but also alleviates environmental concerns [53]. There are still some defects in the membrane reactor: uneven distribution of cells/enzymes on the membrane; steric hindrance changes the spatial configuration of enzymes or covers the active sites; when the pressure is high, the membrane is prone to rupture; the performance of membrane reactors is severely affected by concentration polarization and membrane fouling. In the future, there is potential for the exploration of novel membrane materials, the implementation of process optimization techniques in membrane reactors, and advancement in the development of innovative membrane reactor systems to address the current challenges.

## 3. Immobilized Enzyme

**Figure 3:**
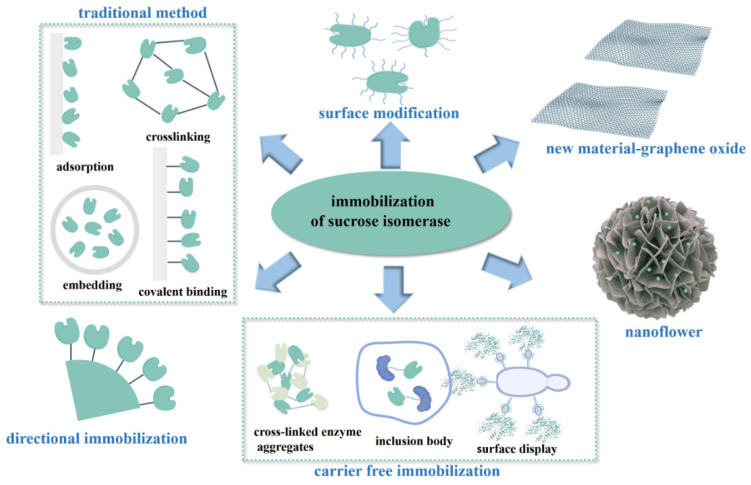
Schematic diagram of immobilized enzyme methods.

Due to the low conversion efficiency of immobilized cells, additional substrate consumption, and autolysis during the reaction process, more and more studies are increasingly interested in the immobilization of sucrose isomerase. Six kinds of immobilized enzyme methods were used to immobilize sucrose isomerase (Figure 3). The four common traditional immobilization methods including adsorption, embedding, covalent binding, and cross-linking were often used to immobilize sucrose isomerase.

In addition to improving the traditional methods of adsorption, embedding, cross-linking, and covalent binding, the researchers also investigated new methods such as graphene oxide as a carrier, or carrier-free cross-linked enzyme aggregates, nanoflowers, and surface display. In recent years, the representative immobilization methods of sucrose isomerase are shown in Table 4.

### 3.1. Innovations in Traditional Immobilizations of Sucrose Isomerase

The traditional methods for the immobilization of enzymes can be divided into two groups: physical and chemical methods. The physical method mainly includes two types: adsorption and embedding, while the chemical method also mainly includes two types: covalent binding and cross-linking [70]. However, there are some problems with the traditional method. Researchers have obtained improved immobilization effects by improving traditional methods.

#### 3.1.1. Adsorption

The adsorption method is a simple and convenient method for immobilizing enzymes. It utilizes the physical interactions between the carrier and enzyme, including van der Waals forces, ionic interactions, and hydrogen bonding, to enable the enzyme to be adsorbed onto the surface of the carrier. The most common inorganic carriers include alumina, diatomaceous earth, porous glass, etc. Organic carriers include chitin, chitosan, cellulose, and alginate. Adsorption is a non-covalent force, and the binding between the carrier and the enzyme is relatively weak. However, it usually does not change the natural structure of the enzyme, which can prevent interference with the active site of the enzyme and maintain high activity of the enzyme [71].

Chen et al. used silicon spheres as carriers to adsorb sucrose isomerase. After 15 cycles of use, the enzyme activity recovery rate of the adsorption method was 55.1%, lower than the 77.9% of the adsorption cross-linking method. This is mainly attributed to the non-covalent adsorption. During the recycling process, continuous washing and centrifugation operations caused the enzyme adsorbed on the surface of the silicon spheres to detach, resulting in a decrease in enzyme activity [58]. Therefore, it is often used as a basic method in combination with other methods.

#### 3.1.2. Embedding

The embedding method involves embedding enzymes in a carrier grid, allowing substrates and products to pass through, reducing enzyme leaching and improving stability, such as organic polymers or sol-gel, which protect enzymes from direct contact with the external environment. The sol-gel method is the most common. This method is simple to operate, with high reaction conditions and mechanical stability, but it also has some drawbacks, such as easy leakage and inactivation of enzymes [72].

Contesini et al. prepared microcapsules using low methoxy pectin and fat (50% butter and 50% oleic acid) to embed sucrose isomerase from *Erwinia* sp. D12. The yield of isomaltulose was only 30%, and after 9 cycles, it was lower than 5%, indicating that the enzyme had been inactivated [54]. When sodium alginate is used to immobilize enzymes, compared to immobilized cells, composite encapsulation is often used due to its larger pore size and easier leakage of enzyme molecules. Zhang et al. used the polyvinyl alcohol sodium alginate embedding method to immobilize sucrose isomerase genes derived from *P. dispersa* UQ68J. The sucrose isomerase produced by *Y. lipolytica* JD exhibited higher enzyme activity after successful embedding. When the substrate sucrose concentration was 650.0 g/L, the yield of isomaltulose was 620.7 g/L, and the yield was 0.96 g/g. The yield of isomaltulose was very high. The sucrose conversion rate remained above 90% in the first 13 cyclic reactions. This method has a high level of enzyme activity; the residual activity remained at 60% after incubation at 50 °C for 4 h, >95% activity was maintained at pH 4.8–7.2, and the yield of isomaltulose was high. However, after the 13 reactions, the conversion rate began to decrease, and it was the lowest in the 18th reaction. The conversion rate was around 30% [55]. Wang et al. also used the polyvinyl alcohol sodium alginate embedding method to immobilize genes derived from the sucrose isomerase produced by *Y. lipolytica* JD, using low-cost beet molasses as a substrate to reduce production costs. After pre-treatment with H_2_SO_4_, centrifugation to remove insoluble substances, and viscosity reduction, under the optimal process conditions, the yield of the eighth batch of isomaltulose was 0.94 g/g, with a purity of 85.8%, a sucrose conversion rate of 97.5%, and a concentration of 446.4 ± 5.5 g/L. After 11 batches, the sucrose conversion rate remained above 94% [56].

#### 3.1.3. Covalent Binding

Covalent binding is the chemical reaction between the side chain amino acids of an enzyme, such as lysine, cysteine, or aspartic acid, and glutamic acid residues to form covalent bonds with the carrier material. Covalent bonds are stable to prevent enzyme leakage from the support matrix, thereby improving the stability of immobilized enzymes and showing good efficiency. However, due to the chemical reaction between the enzyme molecule and the carrier, the enzyme active center may become inactive, leading to a decrease in the activity of the catalyst and requiring additional pre-treatment steps, which are more complex [70,73].

In addition, previous studies have shown that multi-point and multi-subunit covalent immobilization can improve enzyme stability owing to improved enzyme rigidity. Wu et al. used sponge synthesized by ε-poly-L-lysine and gelatin as a carrier to fix sucrose isomerase derived from *E. rhapontici* NX-5. The enzyme formed covalent bonds with the sponge carrier, and glutaraldehyde acted as a connecting arm to achieve higher operational stability. Due to the properties of ε-poly-L-lysine, the sponge has an obvious antibacterial function. The maximum enzyme activity recovery rate in this method was 84.50%, and the corresponding enzyme activity was 71.58 U/g. The yield of isomaltulose was 83.58%, and the purity was 97.3%. The half-life of the immobilized enzyme was 12 days. SI-ε-PL-gelatin exhibited greater pH tolerance and a longer half-life. In the first 156 h of the catalytic process, the yield of isomaltulose remained at almost 80%, and then gradually decreased. At 300 h, the yield of isomaltulose was 48%, and the conversion rate of sucrose was about 50%. After 13 batches of cyclic reactions, the sucrose conversion rate remained above 90%, indicating good operational stability [57].

#### 3.1.4. Cross-Linking

Cross-linking is based on intermolecular reactions, using bifunctional or multifunctional reagents to cross-link enzymes onto the carrier substrate. The covalent bonds formed between enzymes and multifunctional reagents result in a three-dimensional, cross-linked network structure. By covalent bonding, the enzyme is stably immobilized, thereby improving its reusability and stability. However, due to the disorder of the cross-linking reactions, cross-linking can also take place at the active center of the enzyme, leading to a decrease or deactivation of the enzyme activity [74]. The most commonly used cross-linking agent is glutaraldehyde [75].

Cross-linking has the following advantages: simple preparation process, low comprehensive cost, and high mechanical strength. Geng et al. used sodium alginate and sodium carboxymethyl cellulose to fix sucrose isomerase derived from *S. plymuthica* by the embedding method, chitosan-glutaraldehyde adsorption cross-linking method, and cross-linking enzyme aggregate method, respectively. They found that the recovery of the enzyme activity of the chitosan-glutaraldehyde adsorption cross-linking method can reach 70.3%, which is significantly higher than the other two methods. This method has the advantages of easy operation, low comprehensive cost, and high mechanical strength. Under the optimal conditions, the yield of isomaltulose was 87.8%. After 16 consecutive transformations, the product yield remained at 87.52%, rendering it feasible for preliminary adoption in industrial production [59].

### 3.2. Novel Strategies for the Immobilization of Sucrose Isomerase

To receive immobilized enzymes with higher thermal stability, better pH tolerance, and higher immobilization efficiency, researchers also studied new immobilization methods such as carrier-free cross-linked enzyme aggregates, nanoflowers, and directed affinity immobilization of enzyme molecules.

#### 3.2.1. Surface Modification

Through surface modification, the biocompatibility between the enzyme and mesoporous titanium dioxide is improved, and the enzyme load of the carrier is increased, which can improve the operational stability compared with that without surface modification. Wu et al. used mesoporous titanium dioxide with ε-poly-L-lysine to alter its surface charge properties, enabling it to interact with negatively charged sucrose isomerase and thereby enhancing its enzyme loading capacity. Using sucrose as the substrate, mesoporous titanium dioxide and ε-poly-L-lysine-modified mesoporous titanium dioxide were employed as carriers to immobilize sucrose isomerase sourced from *E. rhapontici* NX-5. The research revealed that the enzyme activity of ε-poly-L-lysine-modified mesoporous titanium dioxide (39.41 U/g) was approximately eight times that of mesoporous titanium dioxide (5.04 U/g), and it exhibited greater thermal and pH tolerance. After 5 cycles, mesoporous titanium dioxide exhibited a sucrose conversion rate of less than 90%. By the eighth cycle, the sucrose conversion rate was only 65%, with a half-life of approximately 55 h. On the other hand, ε-poly-L-lysine-modified mesoporous titanium dioxide displayed approximately 95% sucrose conversion after 16 cycles, but it experienced a significant decrease after 19 cycles, with the sucrose conversion rate dropping to 50%. The half-life was approximately 114 h [60].

#### 3.2.2. Carrier-Free Immobilization

With the development of immobilized enzyme technology, carrier-free immobilized enzyme technology has attracted researchers’ attention. Compared with traditional immobilized enzyme technology, this technology does not require expensive carriers, resulting in reduced expenses. It also features smaller particles, a larger specific surface area, heightened enzyme activity, diminished susceptibility to substrate diffusion limitations, and enhanced operational stability [76].

##### Cross-Linked Enzyme Aggregates

The cross-linking enzyme aggregation technology first uses a precipitant to physically aggregate the enzyme, and then adds a cross-linking agent to cross-link the enzyme protein molecules together through covalent bonds [77].

Chen et al. fixed sucrose isomerase from *P. dispersa* UQ68J with polyethylene glycol as a precipitant and dextran aldehyde as a cross-linking agent, and added bovine serum albumin as an additive to improve enzyme activity. With sugarcane juice as a substrate, after 10 cycles of use, the enzyme activity was 91.7%, showing excellent reusability. CLSIAs-BSA displayed high storage stability and maintained 60% of its initial activity after 42 days [61].

##### Inclusion Body

Catalytically active inclusion bodies (CatIBs) are achieved by fusing short peptides or proteins with aggregation characteristics with target proteins or enzymes, which can induce the catalytically active inclusion bodies in host cells [78].

Gao et al. used the N-terminal aggregation sequence of the Ure2 protein as a tag to fuse with sucrose isomerase from *Klebsiella* sp. LX3, demonstrating the feasibility of this method [62]. Ma et al. utilized the tetrameric coiled-coil domain of the cell-surface protein tetrabrachion (TdoT) as a fusion label for the preparation of active inclusion bodies. Compared to the immobilization method of changing enzyme spatial orientation, this method has higher enzyme activity. Compared to traditional enzyme immobilization methods, CatIBs as a self-immobilization method, do not require additional carriers or cross-linking agents [63]. This method can achieve synchronous expression and immobilization without the need for separation and purification, which can reduce workload and preparation costs, and has potential industrial application value [79].

##### Surface Display

Yeast cell surface display technology is a eukaryotic protein expression system that has become an important tool in protein engineering research. It is widely used in protein separation and purification, protein immobilization, protein interactions, and other fields. The basic principle is to fuse the exogenous target protein gene with a specific vector gene sequence and introduce it into yeast cells. After induction of expression, the fused protein contains a yeast wall anchoring structure. The fusion protein can be anchored in the yeast cell wall, thereby immobilizing the target protein on the surface of the yeast cell [80].

Lee et al. used glycosylphosphatidylinositol (GPI)-anchored signal sequences to display the sucrose isomerase gene derived from *Enterobacter* sp. FMB-1 on the surface of *Saccharomyces cerevisiae* EBY100. This method exhibits higher enzyme activity within the temperature range of 35–60 °C. However, as it was the first report of immobilized isomerase bioconversion on the surface of yeast, its isomaltulose yield was highest at a sucrose concentration of 100 mM, only 7.4%. Sucrose can be converted to isomaltulose using this method, but compared to the whole-cell catalysis of *Enterobacter* sp., the conversion rate of this method is very low. Subsequently, the rate at which isomaltulose is converted can be improved by controlling the activity of invertase [66]. Li et al. used cell wall protein Pir 1 as an anchor protein to successfully display sucrose isomerase, a gene derived from *Pantoea dispersa*, on the *Y. lipolytica* CGMCC7326 surface. In 500 g/L sucrose, the highest yield of isomaltulose can reach 93 ± 2%. During the recycling process, after 12 cycles, its relative enzyme activity was 85%, and at the 16th cycle, its relative enzyme activity was 50% [67]. Zheng et al. displayed sucrose isomerase derived from *Pantoea dispersa* on *Yarrowia lipolytica* using the glycosylphosphatidylinositol (GPI) cell wall protein (CWP)-anchored signal sequence, with pre-treated sugarcane molasses as the substrate, and the conversion rate of isomaltulose remained above 85% after 9 cycles of use [68]. Surface display technology can attach enzyme molecules to the cell surface, which can replace the tedious process of purifying and immobilizing enzymes [81]. However, some catalytic reactions require endogenous coenzymes for enzymatic reactions, so this technology limits the types of enzymes. The use of this technology is dependent on the type, structure, and application of the enzymes.

#### 3.2.3. Nanoflowers

The combination of flower-like hybrids to produce nanoflowers has many advantages: simple operation, known mechanisms, mild reaction conditions, and can effectively ensure that the enzyme activity is not destroyed, which has aroused great interest among researchers. Various metals such as calcium, cobalt, copper, manganese, zinc, and so on have been used as nanoflowers to immobilize various enzymes [82].

Meng et al. immobilized sucrose isomerase from *Klebsiella* sp. LX3 using Cu^2+^ nanoflowers. Enzyme activity was maintained for a longer period of time compared to the free enzyme. However, after the first cycle of use, the enzyme activity was only 60% of the initial activity of the enzyme, and only 40% of the enzyme activity was left after six uses. This technology is easy to operate and has great potential for development as a new type of immobilized enzyme technology [64].

#### 3.2.4. Directional Immobilization

Directional immobilization is the process of immobilizing enzyme molecules onto a carrier at predetermined positions, which can maintain the natural conformation of the enzyme and completely expose its active sites [83]. Non-directed immobilization methods usually involve randomly immobilizing enzyme molecules onto a carrier or disorderly cross-linking between enzyme molecules, resulting in significant loss of enzyme activity. However, directed immobilization methods can maintain high enzyme activity and avoid the use of toxic cross-linking agents.

Shi et al. first utilized affinity short peptides to endow the enzyme molecules with directional affinity, followed by photo-cross-linking, to enable the enzyme molecules to be covalently fixed to epoxy resin, using sucrose isomerase derived from *P. dispersa* UQ68J. This method utilizes the photo-cross-linking reaction to covalently fix the enzyme molecules onto the epoxy resin, making it less prone to detachment and improving its reusability. After 11 cycles, the relative enzyme activity of the method can be maintained by more than 50% [69].

### 3.3. Novel Material

Since the new century, the development of materials science has provided a broad development space for immobilized enzyme carriers. The emergence of graphene oxide, magnetic materials, metal–organic frameworks, and membrane materials has provided new opportunities for the development of immobilized enzyme carriers and immobilization technologies [84].

Zhang et al. used graphene oxide as a carrier to immobilize sucrose isomerase from *Erwinia* sp. Ejp617, with a carrier enzyme load of 460 mg/g and an enzyme activity of 727.04 U/mg. After 10 cycles of use, the enzyme activity of this immobilization technology was 80.5%. Compared to free enzymes, the fluctuation of immobilized enzyme activity by pH change was less at pH between 4.0 and 8.0, which showed that the immobilized enzyme has better pH stability. After 45 days of storage, the residual activity still maintained more than 80%, suggesting that the storage stability of the enzyme is further improved after immobilization. There is a non-covalent bonding force between the enzyme and graphene oxide, and hydrogen bonding and van der Waals forces may be the main forces. Therefore, this method can be further improved to further improve efficiency [65].

Recent studies on the immobilization of sucrose isomerases are shown in Table 4. These immobilization methods have both advantages and disadvantages (Table 5). The traditional methods include adsorption, embedding, covalent binding, and cross-linking. These methods have their corresponding disadvantages, such as weak adsorption, easy leakage of embedded enzymes, and serious loss of covalent binding and cross-linking enzyme activity, respectively. Therefore, traditional methods have been improved. New strategies for immobilized sucrose isomerases have been explored. For example, the cross-linked enzyme aggregates have the advantages of simple operation and no carrier, but the loss of enzyme activity is serious. In the future, other strategies can be found to reduce the excessive cross-linking between enzyme molecules, so as to improve enzyme activity. Directional immobilization requires additional carriers to achieve immobilization, but with the development of materials science, better and cheaper carriers will emerge. These new strategies have advantages, but there are also some disadvantages that can be solved with the continuous development of science and technology.

## 4. Expanding the Application of Isomaltulose

Isomaltulose has the characteristics of low hygroscopicity, high stability, low calorie content, low GI value (glycemic index), and pure taste, and has been awarded the GRAS (generally recognized as safe) title by the US FDA. People have a certain tolerance for the intake of many sweeteners, and excessive intake can cause discomfort symptoms such as bloating, bowel ringing, and diarrhea. However, the human body has a high tolerance for isomaltulose, and the daily intake of 50 g will not cause gastrointestinal discomfort and diarrhea. Therefore, after review by the FAO/WHO Joint Food Additives Expert Committee, it has been determined that there is no requirement for the ADI value (acceptable daily intake) of isomaltulose.

Isomaltulose has a wide range of food applications and has attracted the interest of food manufacturers and healthcare professionals [85]. Research has shown that drinking growth milk enriched with isomaltulose has a positive impact on people’s attention and memory [86]. Isomaltulose has prebiotic activity and can stimulate the growth of probiotics, as well as producing short-chained fatty acids and secondary bile acids [87]. Isomaltulose can significantly increase fat oxidation, which is beneficial for long-term weight management and improvement of metabolic risk factors [88]. Isomaltulose can improve fatigue resistance and has the same amount of calories as sucrose. Compared to sucrose, isomaltulose has a slower digestion rate in the small intestine and can provide long-lasting energy, thereby alleviating fatigue [89].

As an ideal substitute for sucrose, isomaltulose has significant commercial and development value, and is widely used abroad, but less widely used domestically. With the improvement of national health awareness and the pursuit of functional foods, isomaltulose has great market potential.

## 5. Conclusions and Outlook

Isomaltulose has broad application prospects, and the most effective enzyme preparation for the production of isomaltulose is sucrose isomerase. In recent years, immobilized enzyme technology has improved the properties of immobilized enzymes, which can reduce production costs and increase industrial production capacity. The characteristics of enzymes are key factors in the immobilization of enzymes, and rational design and directed evolution of enzymes will significantly improve their stability and catalytic activity, greatly improving production efficiency.

The immobilization of sucrose isomerase still faces challenges in industrial production, such as toxic cross-linking agents, expensive carriers, low efficiency, and poor reusability, which require further improvement. Future endeavors should delve into the creation of novel immobilization carriers with enhanced specificity, the formulation of immobilization combinations harnessing complementary benefits, the investigation of fresh affinity peptide labels capable of binding to promising materials, and the development of innovative methodologies, in order to make immobilized sucrose isomerase better applied in industrial production. 

In future research on sucrose isomerase immobilization technology, it is still necessary to make efforts in the following aspects: (1) focusing on the development of a new method that is simple, easy to operate, and universally applicable; (2) searching for a new carrier that is cheap and easy to obtain, easy to separate, has a long service life, and has good mechanical strength; (3) realizing the high catalytic efficiency of the immobilized sucrose isomerase and its continuous and large-scale production through batch reaction and scale-up tests. The use of immobilization technology to improve the catalytic efficiency, enzyme activity, and reusability of sucrose isomerase can make sucrose isomerase better used in food processing, which has broad prospects.

## Figures and Tables

**Table 1:** Synthesis process of isomaltulose and comparison of its physical properties with other by-products.

Synthetic Reaction	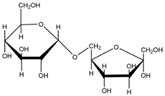 Isomaltulose	SIase 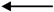	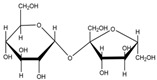 Sucrose	SIase 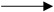	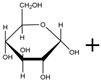 Glucose	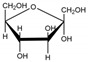 Fructose
Chemical formula	C_12_H_22_O_11_	C_12_H_22_O_11_		C_6_H_12_O_6_		C_6_H_12_O_6_
Molar mass (g/mol)	342.30	342.30		180.16		180.16
Density (g/cm^3^)	1.7	1.77		1.58		1.694
Melting point (°C)	122~124	185–187		146		103~105
Solubility	Easily soluble in water and has a lower solubility in water than sucrose	Soluble in water, insoluble in organic solvents		Soluble in water, slightly soluble in alcohol and acetone, insoluble in ether		Soluble in water, hot acetone, slightly soluble in cold acetone

**Table 2:** Immobilized cell methods for producing isomaltulose in recent years.

Source	Methods	Substrate Conversion Rate	Isomaltulose Yield	Reusability	Refs.
*Serratia* sp.	Alginate embedding		76%	After 35 cycles of use, the yield of isomaltulose is about 60%	[43]
*P. dispersa* UQ68J	Alginate embedding		94%	After 30 cycles of use, the relative enzyme activity remains around 80%	[44]
*P. dispersa* UQ68J	Alginate embedding		90.6%	After 26 cycles, the sucrose conversion rate remains at 83.2%	[45]
*Erwinia* sp. D12	Algae gel and alginate embedding		61.94%	When converted for 72 h, the yield of isomaltulose is 47.86%	[46]
*Erwinia* sp. D12	Alginate glutaraldehyde	71.42%		At 282 h, the yield of isomaltulose is higher than 55%	[47]
*P. rubrum*	Alginate embedding, cross-linking with glutaraldehyde and polyethylene imine	100%	94.5%	After 24 cycles of recycling, the yield of isomaltulose remains above 80%	[48]
*S. plymuthica*	Alginate and gelatin encapsulation, transglutaminase cross-linking		71.04%		[49]
*Erwinia* sp. D12	Alginate and gelatin encapsulation, transglutaminase cross-linking		93.66%		[50]
*P. rubrum*	Sipernat 320 and Eudragit NM		80%		[51]
*S. plymuthicaa* ATCC 15928	Hollow fiber membrane reactor	100%		After continuous operation for 90 days, the activity loss is 11%	[52]
*E. rhapontici* NX-5	Biofilm reactor		92.4%	After 12 cycles, the sucrose conversion rate is above 90%	[53]

**Table 3:** Comparison of different immobilized cell methods.

Methods	Advantages	Disadvantages
Embedding	Non-toxic and harmless, simple operation	Cell leakage
Composite encapsulation	Improved mechanical strength, decreased cell leakage	Reduced the cell contact rate with the substrate and reduced the conversion efficiency
Membrane reactors	Reducing the cost of separation and purification in downstream processing, automated operation	Uneven distribution of cells on the membrane, when the pressure is high, the membrane is prone to rupture, membrane fouling

**Table 4:** Immobilized enzyme methods for producing isomaltulose in recent years.

Source	Methods	Substrate Conversion Rate	Isomaltulose Yield	Reusability	References
*Erwinia* sp. D12	Low methoxy pectin and fat microcapsules embedding		30%	After 9 cycles, the yield of isomaltulose is less than 5%	[54]
*P. dispersa* UQ68J	Polyvinyl alcohol alginate embedding		96%	After 13 batches, the sucrose conversion rate remains above 90%	[55]
*P. dispersa* UQ68J	Polyvinyl alcohol alginate embedding	97.5%	94%	After 11 cycles, the sucrose conversion rate remains above 94%	[56]
*E. rhapontici* NX-5	ε-poly-L-lysine and gelatin		83.58%	At 300 h, the yield of isomaltulose is 48%	[57]
*P. dispersa* UQ68J	Silicon ball glutaraldehyde			After 15 cycles, the relative enzyme activity is 77.9%	[58]
*S. plymuthica*	Chitosan glutaraldehyde		87.8%	After 16 cycles, the yield of isomaltulose remains at 87.52%	[59]
*E. rhapontici* NX-5	ε-Poly L-lysine mesoporous titanium dioxide	Over 95%		After 16 cycles, the sucrose conversion rate is about 95%	[60]
*P. dispersa* UQ68J	Cross-linked enzyme aggregate			After 10 cycles, the relative enzyme activity is 91.7%	[61]
*Klebsiella* sp. LX3	Active inclusion body		80.66 ± 0.82%		[62]
*Klebsiella* sp. LX3	Active inclusion body		82.9 ± 0.92%		[63]
*Klebsiella* sp. LX3	Cu^2+^ nanoflower			After 6 cycles, only 40% of the relative enzyme activity remains	[64]
*Erwinia* sp. Ejp617	Graphene oxide		95.3%	After 10 cycles, the relative enzyme activity is 80.5%	[65]
*Enterobacter* sp. FMB-1	Yeast surface display		7.4%		[66]
*Pantoea dispersa*	Yeast surface display		93 ± 2%	At the 16th cycle of use, its relative enzyme activity is 50%	[67]
*Pantoea dispersa*	Yeast surface display		92.4%	After 9 cycles of use, the conversion rate of isomaltulose remains above 85%	[68]
*P. dispersa* UQ68J	Epoxy resin oriented photo-cross-linking			After 11 cycles, the relative enzyme activity is above 50%	[69]

**Table 5:** Comparison of different immobilized enzyme methods.

Methods	Advantages	Disadvantages
Adsorption	Simple operation	Binding between the carrier and the enzyme is relatively weak
Embedding	Simple to operate, non-toxic, and harmless	Enzyme leakage
Covalent binding	Covalent bonds are stable	Decreased enzyme activity
Cross-linking	Covalent bonds are stable	Decreased enzyme activity
Surface modification	Enhancing enzyme loading capacity	Require additional carriers
Cross-linked enzyme aggregates	Simple to operate, does not require additional carriers	Decreased enzyme activity
Inclusion body	Does not require additional carriers or cross-linking agents	Poor reusability
Surface display	Replace the tedious process of purifying and immobilized enzymes, do not require additional carriers	Dependent on the type, structure, and application of the enzymes
Nanoflower	High specific surface area, simple to operate	Poor structural stability
Directional immobilization	High enzyme activity	Require additional carriers

## Data Availability

No new data were created or analyzed in this study. Data sharing is not applicable to this article.
